# Duration of anticoagulant therapy and VTE recurrence in patients with cancer

**DOI:** 10.1007/s00520-019-4661-3

**Published:** 2019-02-08

**Authors:** Alok A. Khorana, Keith R. McCrae, Dejan Milentijevic, Jonathan Fortier, Winnie W. Nelson, François Laliberté, Concetta Crivera, Patrick Lefebvre, Jeff Schein

**Affiliations:** 1 Department of Hematology and Medical Oncology, Taussig Cancer Institute, Cleveland Clinic and Case Comprehensive Cancer Center, 9500 Euclid Avenue, Cleveland, OH 44195 USA; 2 Hematology and Medical Oncology, Cleveland Clinic Main Campus, 9500 Euclid Avenue, Cleveland, OH 44195 USA; 3 Janssen Scientific Affairs, LLC, 1000 U.S. Route 202 South, Raritan, NJ 08869 USA; 4 Groupe d’analyse, Ltée, 1190, avenue des Canadiens-de-Montréal, Tour Deloitte, Suite 1500, Montréal, QC H3B 0G7 Canada

**Keywords:** Anticoagulant, Cancer, Venous thromboembolism, Recurrence, Duration of therapy

## Abstract

**Purpose:**

Anticoagulant therapy for at least 3–6 months is currently recommended for treatment of venous thromboembolism (VTE) in patients with cancer, but the optimal duration of treatment is unknown. This study examines the association between the duration of anticoagulation treatment and VTE recurrence in cancer patients.

**Methods:**

The Humana claims database was used to identify newly diagnosed cancer patients who had their first VTE diagnosis between January 1, 2013, and May 31, 2015, and initiated injectable or oral anticoagulant therapy. Follow-up was calculated from the index treatment initiation to the end of eligibility or end of data (June 2015). VTE recurrence was defined as a hospitalization with a primary diagnosis of VTE. Cox proportional hazards models were used to evaluate the risk of VTE recurrence by duration of therapy in patients who discontinued therapy.

**Results:**

The study included 1158 patients. Compared to patients treated for 0 to 3 months, VTE recurrences were significantly lower among patients treated for 3 to 6, or over 6 months. After adjustment for baseline characteristics, patients treated for 3 to 6 months (HR [95%CI], 0.53; 0.37–0.76) and more than 6 months (HR [95%CI], 0.48; 0.34–0.68) were still significantly less likely to have VTE recurrences compared to patients treated for 0 to 3 months (both *p* < 0.01). Findings were similar using a VTE event definition that included outpatient visits.

**Conclusions:**

Among newly diagnosed cancer patients with VTE, anticoagulant therapy lasting more than 3 months was associated with a lower risk of VTE recurrence.

**Electronic supplementary material:**

The online version of this article (10.1007/s00520-019-4661-3) contains supplementary material, which is available to authorized users.

## Introduction

Venous thromboembolism (VTE) is the second leading cause of morbidity and mortality in patients with cancer [1]. It is estimated that the annual incidence of VTE is approximately 1 out of 200 in a population of cancer patients [2]. When compared to the general population, patients with cancer are associated with up to 6.5-fold higher risk of VTE [3, 4]. Furthermore, the risk of recurrence after a first episode of VTE is higher in cancer patients than in those without underlying malignancy [5].

Current guidelines recommend anticoagulation treatment with low-molecular-weight heparin (LMWH) monotherapy for at least 3 to 6 months for treatment and secondary prophylaxis in patients with cancer [6, 7]. Treatment beyond the initial 6 months should also be considered for patients with metastatic disease or those receiving chemotherapy. In practice, many patients with cancer are treated for less than the recommended 3 to 6 months, and more than half of patients are not treated with LMWH [8–11].

In the overall patient population with VTE, longer duration of therapy (DOT) has been associated with a reduced risk of VTE recurrence, specifically for patients treated for at least 3 months [12–14]. However, studies regarding the optimal duration of anticoagulant therapy in patients with cancer are lacking. In this analysis, we examined the association between the duration of anticoagulation and rates of VTE recurrence and major bleeding in cancer patients.

## Methods

### Data source

Medical and pharmacy claims from the Humana database from January 2007 to May 2015 were used to conduct the analysis. The Humana database includes over 18 million covered lives of commercial and Medicare members in all census regions in the USA but predominantly in the Midwest and South Regions. Over 9 million members have both medical and pharmacy coverage. The present study used data elements such as demographics, enrollment history, inpatient and outpatient claims, emergency-department visits, and pharmacy claims for commercial and Medicare Advantage Part D (MA-PD) members. Data were de-identified and data collection complied with the requirements of the Health Insurance Portability and Accountability Act (HIPAA).

### Study design

A retrospective cohort design was used to evaluate the association between the duration of anticoagulation and VTE recurrence and major bleeding events. Newly diagnosed patients with cancer were identified with at least one inpatient stay or two outpatient visits with a diagnosis of cancer, and with a first VTE (index VTE) after 2013. The index VTE had to occur after a patient’s first cancer diagnosis (a 30-day window before the cancer diagnosis was allowed since a VTE can be an early sign of cancer). Patients with one or more dispensing of an anticoagulant agent (LMWH, warfarin, or rivaroxaban) within 7 days after their VTE diagnosis were selected. Other anticoagulant treatments were not included due to low utilization and short follow-up time. Treatment discontinuation was defined as a gap of more than 60 days between the end of the days of supply and the next dispensing of the index therapy. Patients with a prior VTE diagnosis or anticoagulant treatment before the index cancer and patients with a prior dispensing of an anticoagulant before the index VTE were excluded from the study.

Based on the DOT of the first anticoagulant agent received, patients were classified into one of the following cohorts: DOT 0 to 3 months, DOT 3 to 6 months, and DOT over 6 months. The observation period spanned from the date of the first anticoagulant dispensing to the end of insurance eligibility or the end of data availability. During this observation period, outcomes were assessed when patients were on and off the initial treatment.

### Study endpoints

The main endpoint was first VTE recurrence, defined conservatively as a hospitalization with a primary diagnosis of VTE. As a sensitivity analysis, a broader definition of VTE recurrence, in which both inpatient and outpatient VTE events were included, was assessed. Outpatient VTE events were identified with diagnoses and radiology imaging procedures for VTE during outpatient visits. As a secondary endpoint, major bleeding events were identified based on a primary diagnosis ICD-9-CM code for any bleeding at a gastrointestinal, genitourinary, cerebral, or other relevant site as identified by a validated algorithm developed by Cunningham et al. designed to identify hospitalizations related to bleeding [15]. A major bleeding event was also identified by the algorithm if a patient had a primary diagnosis for selected conditions such as chronic or unspecified ulcer with perforation, esophagitis, and acute post-hemorrhagic anemia, and a secondary diagnosis of bleeding at one of the bleeding sites identified above. The use of bleeding diagnoses has shown a positive predictive value of 89 to 99% in Cunningham’s validation study [15].

### Statistical analysis

Patient characteristics were summarized by treatment cohorts (i.e., DOT 0 to 3 months, DOT 3 to 6 months, and DOT over 6 months). Descriptive statistics, including mean (standard deviation [SD]) for continuous data and relative frequency for categorical data, were generated to describe the baseline characteristics of the different treatment cohorts. Patient characteristics were assessed during the 6-month baseline period prior to the index VTE. The type of index cancer was also reported and may have occurred before the 6-month baseline period.

Rates of VTE recurrence and bleeding events were calculated as the number of events per 100 patient-years, and were truncated at the first event for each endpoint. Therefore, the observation period for at risk for VTE recurrence and major bleeding may be different for a given patient. Cox proportional hazards models adjusting for potential differences in patient characteristics between the different cohorts were used, controlling for age, sex, region, CCI, type of index VTE (PE, DVT, or both), hospitalization for the index VTE, time from first diagnosis of cancer to first VTE, type of cancer, and antineoplastic agent received during the baseline period, to compare the risk of VTE recurrence and major bleeding between cohorts.

A limitation of the design was that patients in the DOT 3 to 6 months and DOT over 6 months cohorts had to stay alive long enough to be in those cohorts, thus biasing these patients to be healthier and potentially to have fewer VTE and major bleeding events (i.e., immortal time bias). To assess the impact of this bias, a second sensitivity analysis was done in which all study analyses were repeated among only patients who had at least 9 months of data after the index date.

## Results

### Patient characteristics

A total of 1158 newly diagnosed patients with cancer who developed VTE and were treated with anticoagulant agents were identified and stratified into three cohorts based on the duration of their index anticoagulant therapy: 629 in the DOT 0 to 3 months, 244 in the DOT 3 to 6 months, and 285 in the in the DOT over 6 months cohorts. Patients were observed for a mean of 13.7 months after the initiation of therapy. Mean follow-up was shorter in patients treated for a shorter time: 10.9 months in the DOT 0 to 3 months cohort, 14.9 in the DOT 3 to 6 months cohort, and 19.0 in the DOT over 6 months cohort. More patients in the DOT 0 to 3 months cohort were treated with injectable LMWH 293 (47%) than with an oral anticoagulant: warfarin (29%) or rivaroxaban (24%). The number of patients treated with LMWH decreased in the longer DOT cohorts: 49 (20%) in the DOT 3 to 6 months cohort and 34 (11.9%) in the DOT over 6 months cohort, respectively (Table 1).

**Table 1:** Patient demographics and clinical characteristics

	DOT 0 to 3 months (*N* = 629)	DOT 3 to 6 months (*N* = 244)	DOT over 6 months (*N* = 285)
Age, mean (SD) [median]	71.9 (9.7) [72.0]	71.7 (11.2) [72.0]	73.4 (8.5) [73.0]
Gender, female, *n* (%)^1^	307 (48.8)	131 (53.7)	141 (49.5)
Region, *n* (%)			
South	369 (58.7)	145 (59.4)	160 (56.1)
Midwest	174 (27.7)	69 (28.3)	89 (31.2)
Northeast	16 (2.5)	4 (1.6)	6 (2.1)
West	70 (11.1)	26 (10.7)	30 (10.5)
Race/ethnicity, *n* (%)			
White	498 (79.2)	191 (78.3)	218 (76.5)
Black	72 (11.4)	21 (8.6)	48 (16.8)
Hispanic	4 (0.6)	5 (2.0)	0 (0.0)
Other	11 (1.7)	7 (2.9)	2 (0.7)
Unknown	44 (7.0)	20 (8.2)	17 (6.0)
Time from cancer to first VTE, *n* (%)			
Less than 6 months	349 (55.5)	126 (51.6)	135 (47.4)
6 months to 1 year	79 (12.6)	31 (12.7)	45 (15.8)
More than 1 year	201 (32.0)	87 (35.7)	105 (36.8)
Type of index VTE, *n* (%)			
PE	159 (25.3)	57 (23.4)	82 (28.8)
DVT	389 (61.8)	164 (67.2)	152 (53.3)
PE and DVT	81 (12.9)	23 (9.4)	51 (17.9)
Type of anticoagulant therapy, *n* (%)			
LMWH	293 (46.6)	49 (20.1)	34 (11.9)
Warfarin	185 (29.4)	119 (48.8)	169 (59.3)
Rivaroxaban	151 (24.0)	76 (31.1)	82 (28.8)
Type of primary cancer^2^, *n* (%)			
Solid cancer	558 (88.7)	212 (86.9)	251 (88.1)
Lung	106 (16.9)	27 (11.1)	36 (12.6)
Prostate	69 (11.0)	37 (15.2)	40 (14.0)
Breast	69 (11.0)	35 (14.3)	50 (17.5)
Colorectal	78 (12.4)	33 (13.5)	40 (14.0)
Other solid cancer	236 (38)	80 (32.8)	85 (29.8)
Hematologic cancer	73 (11.6)	34 (13.9)	36 (12.6)
Risk for VTE			
Very high risk^3^	56 (8.9)	11 (4.5)	10 (3.5)
High risk^4^	214 (34.0)	73 (29.9)	90 (31.6)
Antineoplastic use at baseline^5^, *n* (%)	87 (13.8)	42 (17.2)	41 (14.4)
Quan-Charlson comorbidity index^5^, mean (SD) [median]	4.8 (3.1) [5.0]	4.7 (2.7) [5.0]	4.4 (2.9) [4.0]
Selected baseline comorbidities^5^, *n* (%)			
Hypertension	435 (69.2)	184 (75.4)	214 (75.1)
COPD	196 (31.2)	60 (24.6)	72 (25.3)
Diabetes	172 (27.3)	73 (29.9)	91 (31.9)
Congestive heart failure	92 (14.6)	34 (13.9)	35 (12.3)
Liver diseases	98 (15.6)	43 (17.6)	26 (9.1)
Obesity	94 (14.9)	34 (13.9)	39 (13.7)
Atrial fibrillation/flutter	46 (7.3)	17 (7.0)	13 (4.6)
Stroke/TIA	26 (4.1)	6 (2.5)	19 (6.7)

*SD*, standard deviation; *VTE*, venous thromboembolism; *DVT*, deep venous thrombosis; *PE*, pulmonary embolism; *COPD*, chronic obstructive pulmonary disease; *TIA*, transient ischemic attack; *LMWH*, low-molecular-weight heparin

^1^The denominator of all percents is the respective cohort

^2^Not mutually exclusive

^3^Stomach, pancreas, or brain tumor

^4^Lung, lymphoma, gynecologic, bladder, testicular, or renal cancer

^5^Evaluated during the 6-month baseline period

Patients in the three cohorts were similar in their demographic characteristics, with median age ranging from 72 to 73 years. The DOT 0 to 3 months cohort tended to have more patients with a cancer type associated with a higher risk of developing a VTE: 8.9% of patients with very high-risk cancer types (stomach, pancreas, or brain tumor) and 34.0% of patients with high-risk cancer types (lung, lymphoma, gynecologic, bladder, testicular, or renal cancer). PE, with or without a concomitant DVT diagnosis, was more frequent in patients treated for a longer period: accounting for 133 (46.7%) of the type of index VTE in the DOT over 6 months cohort, while accounting for 240 (38.2%) and 80 (32.8%) in the DOT 0 to 3 months and the DOT 3 to 6 months cohorts, respectively (Table 1).

### VTE recurrence

In the overall study population, VTE recurrence occurred in 233 (20.1%) patients, 134 (11.6%) while on therapy, and 99 (8.5%) after anticoagulant discontinuation (Table 2). The VTE recurrence rate for the entire observation period during and after treatment was 20.3 per 100 patient-years. The VTE recurrences were over two-fold higher in the cohort of patients treated for 0 to 3 months (31.9) compared to cohorts treated for 3 to 6 months (13.5) and over 6 months (11.0). More than half of patients had their VTE recurrence during their initial therapy (134/233; 58%). The percentage of patients with a VTE recurrence while on treatment was similar across cohorts: 12.7% in the DOT 0 to 3 months cohort, 8.6% in the DOT 3 to 6 months cohort, and 13.5% in the DOT over 6 months cohort. As expected, using the broader definition of a VTE event as a sensitivity analysis resulted in a higher proportion of patients with a VTE recurrence. Specifically, in the general population, 32.6% of patients had a VTE recurrence and the rate recurrence was 37.4 per 100 patient-years.

**Table 2:** Rate of VTE recurrences and major bleeding events—stratified by duration of therapy^1^

	All treated patients (*N* = 1158)	DOT 0 to 3 months (*N* = 629)	DOT 3 to 6 months (*N* = 244)	DOT over 6 months (*N* = 285)
Follow-up (months)^2^ mean ± SD [median]	13.7 ± 7.6 [12.3]	10.9 ± 7.1 [8.8]	14.9 ± 6.9 [13.4]	19.0 ± 6.0 [18.4]
VTE recurrence^3^, *n* (%)	233 (20.1)	152 (24.2)	37 (15.2)	44 (15.4)
On index AC therapy, *n* (%)	134 (11.6)	80 (12.7)	21 (8.6)	33 (13.5)
Post index AC period, *n* (%)	99 (8.5)	72 (11.4)	16 (6.6)	11 (3.9)
Rate (per 100 patient-years)	20.3	31.9	13.5	11.0
Bleeding events^4^, *n* (%)	141 (12.2)	89 (14.1)	15 (6.1)	37 (13.0)
On index AC therapy, *n* (%)	81 (7.0)	50 (7.9)	9 (3.7)	22 (7.7)
Post index AC period, *n* (%)	60 (5.2)	39 (6.2)	6 (2.5)	15 (5.3)
Rate (per 100 patient-years)	11.5	17.5	5.1	8.9

*RVTE*, recurrence of VTE; *LMWH*, low-molecular-weight heparin

^1^Duration of therapy was calculated from the first anticoagulant dispensing to treatment nonpersistence (i.e., a gap of more than 60 days between the end of the days of supply of a dispensing and the next dispensing of the index therapy)

^2^From the index treatment initiation to the end of eligibility or end of data (June 2015)

^3^A VTE recurrence was identified if a patient had a primary diagnosis of VTE during a hospitalization

^4^Major bleeding events were identified using a validated algorithm developed by Cunningham et al.

**Fig. 1:**
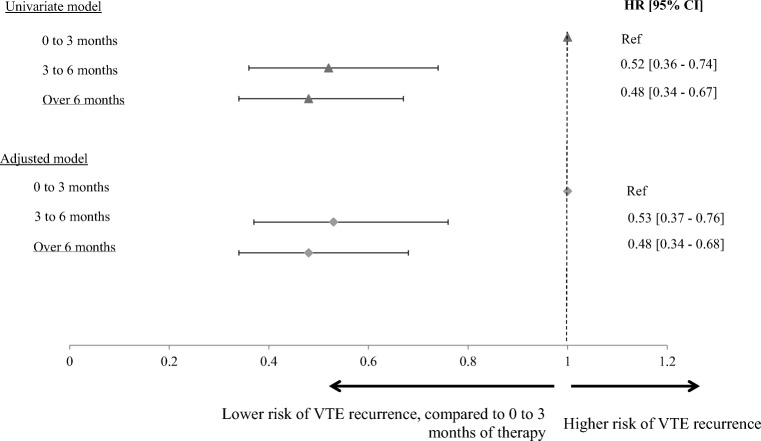
Cox proportional hazard model for time to recurrence

Compared to patients treated for 0 to 3 months, the risk of VTE recurrence was reduced by half for patients treated for more than 3 months; the hazard ratio (HR) was 0.52 [0.36–0.74] for the patients treated for 3 to 6 months and 0.48 [0.34–0.67] for patients treated for more than 6 months (Fig. 1). The multivariate cox model shows similar results, with an HR of 0.53 [0.37–0.76] and 0.48 [0.34–0.68], for the DOT 3 to 6 months and DOT over 6 months cohorts, respectively. The sensitivity analysis identifying VTE events from both inpatient and outpatient visits showed similar results with HRs of 0.67 [0.52–0.88] for 3 to 6 months and 0.55 [0.42–0.71] for patients treated for more than 6 months.

### Bleeding events

A total of 141 (12.2%) patients were identified with a major bleeding event over the entire follow-up period. More than half, 89 (63%) of the major bleeding events occurred in patients treated for less than 3 months, and most of them occurred while the patient was on therapy. The overall bleeding rate was 11.5 per 100 patient-years (Table 2).

### Sensitivity analysis

In order to assess the impact of immortal time bias, all analyses were repeated among only patients with at least 9 months of follow-up data (Appendices 1 and 2). The rates of VTE recurrence and major bleeding were lower across all patients (VTE recurrence, 13.9 per 100 patient-years; major bleeding, 8.6 per 100 patient-years), the DOT 0 to 3 months cohort, and the DOT 3 to 6 months cohort as compared to the patients in the main analyses. The rates were similar in the DOT over 6 months cohort. While the VTE rates were overall lower in the sensitivity analysis results, the HRs based on the unadjusted and adjusted models were very similar to the main analysis results. Compared to patients treated for 0 to 3 months, the multivariate Cox model showed that VTE recurrence was similarly reduced by half for patients treated between 3 and 6 months, with a HR of 0.51 [0.32–0.82]. It was reduced by a third for patients treated for more than 6 months, with a HR of 0.61 [0.41–0.89].

## Discussion

This real-world analysis of treatment duration for VTE showed that most patients were treated for less than 3 months despite guideline recommendations for a minimum of 3 to 6 months. Results from this study showed that less than 3 months of anticoagulant therapy was associated with up to two-fold higher risk of VTE recurrence during and after anticoagulant therapy. The benefit of anticoagulation in patients with cancer outweighs the risk of major bleeding as the rates of VTE recurrence were up to 40% higher than the rates of major bleeding across all cohorts.

It is well documented that the risk of VTE recurrence is significantly higher in cancer patients than in the overall population [16]. The risk of VTE recurrence at 12 months has been estimated at 13 to 21% for patients with cancer [5, 9]. In the current study, 20.3% of patients experienced a VTE recurrence over 14 months of follow-up which is consistent with previous findings. The percent increased to 32.6% when a VTE recurrence was additionally identified using both diagnosis codes and scans. Out of the 20.3% of patients identified with the conservative VTE definition, 11.6% had a recurrent event during anticoagulation and 8.5% had an event after anticoagulant discontinuation. These results are similar to clinical trials data, where 8.0% and 15.8% of patients experienced a VTE recurrence over the 120 days of therapy in the CLOT study [17] and 7.2% and 10.5% of patients over 150 days of therapy in the CATCH trial [18]. The rate of VTE recurrence of 20.3 events per 100 patient-years in the current study is generally consistent with rates of VTE reported in patients with cancer. In a meta-analysis of randomized trials, the rate of VTE recurrence was estimated to be 13.9 per 100 patient-years for patients treated with vitamin K antagonist (VKA) and 7.1 per 100 patient-years for patients treated with LMWH [19]. A higher rate of VTE recurrence was reported in an observational study, with a rate of 30.0 per 100 patient-years [5].

To our knowledge, no study has compared the relationship between the duration of anticoagulant therapy and the risk of VTE recurrence in a cancer population. Nonetheless, studies in the general VTE patient population found that longer anticoagulant treatment reduced the risk of VTE recurrence. A meta-analysis of five randomized trials comparing therapy for less than 3 months (4 to 6 weeks) to 3 to 6 months of therapy found a relative risk of 1.83 after the treatment discontinuation for patients treated less than 3 months when compared to patients treated for 3 to 6 months. No significant difference was observed in risk of recurrence in patients treated for 3 to 6 months compared to patients treated for 6 to 12 months [14]. The current study found a similar risk reduction in cancer patients treated for 3 to 6 months compared to those treated for less than 3 months.

While the risk of VTE recurrence is higher in cancer patients, the risk of bleeding complications is also higher in these patients; the duration of anticoagulant therapy to reduce the risk of VTE recurrence needs to be balanced with the increased risk of bleeding [20]. In the current study, 12.2% of patients were identified with a major bleeding event during the follow-up period: 7.0% while on index anticoagulant therapy and 5.2% post index therapy. These percentages are higher than the 2 to 6% observed in clinical trials [17, 18]. Patients included in the current study were considerably older, with a median age of 72 years, and many of the patients had diagnoses related to bleeding risk factors [21]: hypertension (over 70%), congestive heart failure (14%), and diabetes (30%). The overall bleeding rate of 11.5 per 100 patient-years observed in the current study is in the range of previously reported results in other observational studies in patients with cancer. Kleinjan reported a rate of 6.8 per 100 patient-years for patients treated with LMWH and 3.6 per 100 patient-years for patients treated with VKA [22], while Masseria reported a higher rate of 15.9 per 100 person-years among patients with cancer [23]. In the current study, we observed that 43% of the major bleeding events occurred after the discontinuation of initial therapy, highlighting the risk of bleeding among cancer patients for reasons other than anticoagulants. The rate of major bleeding was already reported in cancer patients regardless of the use of anticoagulant therapy, ranging from 4.2 to 9.6% for patients with cancer without VTE [24, 25].

The current guidelines recommend anticoagulation for at least 3 to 6 months for the treatment of VTE and as secondary prophylaxis in patients with cancer, but these guidelines are not regularly adhered to [6, 7]. Patients with cancer are in an elevated prothrombotic state and are at high risk for first and recurring VTE. In a population-based cohort study of patients with active cancer, the cumulative VTE recurrence rates at 3 months, 6 months, and 1 year were 18.0%, 21.4%, and 26.7%, respectively, after an initial diagnosis of VTE [26]. The highest proportion of patients who experienced a recurrence had one between 0 and 3 months. Similarly, in this study, the highest VTE recurrence of 24.2% (12.7% during and 11.4% post treatment) was observed in the DOT 0 to 3 months cohort. Furthermore, only 7.9% of patients in the DOT 0 to 3 months cohort had on-treatment major bleeding; therefore, the short treatment duration, or early discontinuation, cannot be fully due to adverse events. Other factors, including treatment cost, route of administration, and patient education on VTE and lifestyle preference, may have played a role in the early discontinuation. Poor adherence to treatment leading to discontinuation may also explain the higher utilization of LMWH in the DOT 0 to 3 months, a finding that likely contributed to the higher rate of VTE recurrence observed in this cohort [27]. These results, previously reported, further support the notion that the real-world efficacy of LMWH is reduced compared with oral anticoagulant [25]. Therefore, an informed treatment decision with an emphasis on continue anticoagulation rather than using only recommended LMWH may be more important to mitigate premature discontinuation. The current study shows significant benefit over the risk associated with anticoagulation treatment lasting more than 3 months.

This retrospective cohort analysis has certain limitations. First, the study design was such that patients in the DOT 3 to 6 months and over 6 months cohorts had to be observed in the data longer than patients in the DOT 0 to 3 months cohort, thus potentially biasing our results. A sensitivity analysis among only patients with at least 9 months of follow-up data resulted in similar findings to the main analysis, thus validating our study results. Second, in spite of information accuracy and completeness required by administrative databases for payment purposes, billing inaccuracies and missing data may still occur. Third, the analysis assumed that all the medications supplied were used by the patients, which may not always be the case. We hypothesize that due to high out-of-pocket costs, patients would only fill a prescription they intended to use. If there were unused prescriptions, the impact would be an overestimation of patients’ duration of anticoagulant therapy, and thereby reduce the between-group difference (biased to the null). Fourth, the observational design was susceptible to biases such as information or classification bias (e.g., identification of false-positive VTE events). It is also possible that VTE events were under-coded (i.e., false-negative). However, it is important to note that defining VTE events based on inpatient visits and then on both inpatient and outpatient visits as a sensitivity analysis resulted in similar findings. Finally, like all observational studies, adjustments in the multivariate analyses could only account for observable factors. Despite these limitations, the current study has several strengths, including reliance on the real-world utilization of anticoagulant therapies in patients with cancer.

Anticoagulant therapy for VTE lasting longer than 3 months is associated with over 50% reduction in risk of VTE recurrence in patients with cancer. The incidences of VTE recurrence in patients treated less than 3 months are three times higher than the incidence of major bleeding during and after treatment, indicating the need for longer anticoagulation without increasing the risk of bleeding. Furthermore, the rate of major bleeding for patients treated for more than 6 months was similar as that for patients treated for 3 to 6 months. These findings should help inform patients’ and oncologists’ decisions over benefits and risks regarding duration of anticoagulation therapy in patients with cancer.

## Electronic supplementary material

ESM 1 (DOCX 19 kb)
