# Supplementary material for: Duration of anticoagulant therapy and VTE recurrence in patients with cancer
Source: Support Care Cancer. 2019 Feb 8;27(10):3833–40. doi: 10.1007/s00520-019-4661-3 (PMC6726708; doi:10.1007/s00520-019-4661-3)
Supplement: Supplementary file 1 — (DOCX 19 kb) [file 520_2019_4661_MOESM1_ESM.docx]

**Appendix 1.** Rate of VTE Recurrences and Major Bleeding Events ‒ Stratified by Duration of Therapy^1^ among Patients with ≥9 Months of Follow-Up

|  | All treated patients | DOT 0 to 3 months | DOT 3 to 6 months | DOT over 6 months |
| --- | --- | --- | --- | --- |
|  | **(N=766)** | **(N=303)** | **(N=182)** | **(N=281)** |
| Follow-up (months)^2^ mean ± SD [median] | 17.8 ± 6.1 [16.9] | 16.7 ± 6.1 [15.3] | 17.5 ± 6.1 [16.1] | 19.1 ± 5.9 [18.6] |
|  |  |  |  |  |
| VTE recurrence^3^, n (%) | 137 (17.9) | 69 (22.8) | 24 (13.2) | 44 (15.7) |
| On index AC therapy, n (%) | 84 (11.0) | 37 (12.2) | 14 (7.7) | 33 (18.1) |
| Post index AC period, n (%) | 35 (4.6) | 24 (7.9) | 7 (3.8) | 4 (1.4) |
| Rate (per 100 patient-years) | 13.9 | 19.6 | 10.0 | 11.1 |
|  |  |  |  |  |
| Bleeding events^4^, n (%) | 90 (11.7) | 46 (15.2) | 7 (3.8) | 37 (13.2) |
| On index AC therapy, n (%) | 56 (7.3) | 29 (9.6) | 5 (2.7) | 22 (7.8) |
| Post index AC period, n (%) | 34 (4.8) | 17 (6.2) | 2 (1.1) | 15 (5.8) |
| Rate (per 100 patient-years) | 8.6 | 12.4 | 2.7 | 8.9 |

RVTE=Recurrence of VTE; LMWH: Low-molecular-weight heparin

**Notes:**

1. DOT was calculated from the first anticoagulant dispensing to treatment nonpersistence (i.e., a gap of more than 60 days between the end of the days of supply of a dispensing and the next dispensing of the index therapy).

2. From the index treatment initiation to the end of eligibility or end of data (June 2015).

3. A VTE recurrence was identified if a patient had a primary diagnosis of VTE during a hospitalization.

4. Major bleeding events were identified using a validated algorithm developed by Cunningham et al.
